# The association of catastrophizing with quality-of-life outcomes in patients with irritable bowel syndrome

**DOI:** 10.1007/s11136-017-1554-0

**Published:** 2017-03-21

**Authors:** LeeAnne B. Sherwin, Emily Leary, Wendy A. Henderson

**Affiliations:** 1 Sinclair School of Nursing, University of Missouri, Columbia, MO USA; 2 Biostatistics & Research Design Unit, School of Medicine, University of Missouri, Columbia, MO USA; 3 Biobehavioral Branch, National Institute of Nursing Research, National Institutes of Health, DHHS, Bethesda, MD USA

**Keywords:** Irritable bowel syndrome, Catastrophizing, Quality of life, Illness representations

## Abstract

**Background:**

Catastrophizing is a cognitive process characterized by a propensity to concentrate on and magnify the value of an actual or anticipated painful stimulus and negatively assesses one’s ability to cope. Catastrophizing is an important predictor of pain-related outcomes. A cornerstone symptom of irritable bowel syndrome (IBS) is abdominal pain or discomfort. Also individuals with IBS have been reported to have a tendency to catastrophize. In a sample of individuals who suffer from IBS, we hypothesized that those individuals who catastrophize (catastrophizers) would have worse outcomes as compared to those who do not catastrophize (non-catastrophizers).

**Methods:**

One hundred and one adults with IBS (79% female, mean age 42 years, 97% Caucasian) were recruited from outpatient clinics and data were collected through self-report measures. Catastrophizing was measured with the catastrophizing subscale of the Coping Strategies Questionnaire, illness representations were measured with The Revised Illness Perception Questionnaire (IPQ-R), psychological distress was measured with the Brief Symptom Inventory 18 (BSI-18), and health-related quality of life was measured using the Irritable Bowel Syndrome-Quality of Life (IBS-QOL) measure. Descriptive statistics, correlations, and multiple linear regression analyses were completed to describe participants, the associations of the variables of interest, and the unique relationship between psychosocial variables and HRQOL.

**Results:**

Overall, participants reported poor HRQOL (*M* = 63.32, range 0–100). Catastrophizers differed significantly on IBS-QOL from non-catastrophizers (*M* = 48.98 vs. non-catastrophizers *M* = 78.53; *p* < 0.001), BSI-18 (*M* = 21.35 vs. non-catastrophizers *M* = 6.76; *p* < 0.001), and IPQ-R, specifically the consequences (*M* = 21.75 vs. non-catastrophizers *M* = 17.20; *p* < 0.001) and emotional representations (*M* = 20.90 vs. non-catastrophizers *M* = 15.43; *p* < 0.001). Catastrophizing was positively correlated with the consequences (*r* = .54; *p* < 0.01) and emotional representations (*r* = .65; *p* < 0.01) and negatively correlated with total HRQOL (*r* = −0.76; *p* < 0.01**)**.

**Conclusion:**

The findings indicated that participants who catastrophized reported worse psychosocial and functional outcomes. Thus, catastrophizing, *in addition to psychological distress variables*, may be an important factor to address in optimizing health outcomes in individuals with IBS. In addition, illness perceptions were strongly related to catastrophizing and HRQOL; assessment and integration of illness perceptions as well as catastrophizing into the management of individuals who suffer with IBS may maximize the health outcomes.

## Introduction

Irritable bowel syndrome (IBS) is a highly prevalent chronic gastrointestinal disorder designated by abdominal pain or discomfort and associated bowel habit changes [1]. The symptoms, hardships, and impairments characterize IBS rather than organic abnormalities. Dilemmas concerning diagnosis and the variability of symptoms combined with the complex interaction of factors that impact biological, psychological, and social aspects for an individual with IBS contribute to treatment challenges [2]. The biopsychosocial model presents the relationships between psychosocial factors (life stress, psychological state, social support, and coping), physiological factors (motility, sensation, inflammation, and microbiome), symptoms and behaviors, and clinical outcomes (quality of life, doctor visits, functioning, and medical treatments).

Impairment of quality of life and maladaptive coping have been found in those who suffer from irritable bowel syndrome [3, 4]. Additionally, Rutter and Rutter [3] found that adaptive coping can enhance outcomes such as quality of life and satisfaction with health. Likewise, coping was found to mediate the relationship between illness representations and outcomes. Maladaptive coping, such as catastrophizing, has been found to be endorsed by individuals with IBS [5]. The construct catastrophizing is broadly perceived as an exaggerated negative “mental set” that comes to be when an individual is experiencing pain or is anticipating a pain experience [6]. It is a “method of cognitively coping that is characterized by negative self-statements and overly negative thoughts and ideas about the future” [7]. Catastrophizing has been associated with increased pain severity, disability and functional limitations, decreased quality of life, and worsening disease activity as measured by physiologic indices in those with rheumatoid diseases [8]. Specific to IBS, van Tilburg et al. [5] observed a direct association between catastrophizing and IBS severity; in addition, catastrophizing was found to mediate the relationship between anxiety and IBS severity. Individuals with IBS often experience comorbid psychiatric disorders such as anxiety, depression, and somatization [9, 10]. These psychological comorbidities have been reported to have a negative impact on IBS symptom severity [5] and health-related quality of life [11] and are strong predictors of psychological functioning [12, 13]. Accordingly, the intrinsic perceptions or illness representations of individuals are important roots to their IBS.

The common sense model (CSM) of illness representations was developed by Leventhal and colleagues [14] and expanded to include parallel emotional representations [15]. Illness representations are classified into the following categories: identity (the label and symptoms associated with an illness), timeline (beliefs regarding the time of development and duration), consequences (the ramification of the illness on the psychological, social, and physical functioning), cause (the individual’s belief about potential cause(s) of their illness), control (the belief regarding the amount of control the individual has regarding their ability to control symptoms and the belief in the provider to intercede and influence symptoms), and emotional representation (the negative impact of their illness on their emotional well-being). According to the CSM, illness representations and coping influence outcomes.

Research examining the role of catastrophizing and illness representations in IBS is limited. Therefore, the aim of the present study was to investigate the relevance of catastrophizing to patient-reported illness representations, psychological distress, and health-related quality of life. Specifically, the main focus of the study was to investigate whether there were reporting differences between catastrophizers and non-catastrophizers.

## Methods

### Study design

Self-reported questionnaires were used in this cross-sectional descriptive study. Participants were recruited by physician referral and by advertisement in community-based gastrointestinal practices located in Idaho and Connecticut. The study was approved by the institutional review board of Oregon Health & Science University with a waiver of consent. The study population consisted of patients with an active diagnosis of IBS consistent with Rome III criteria. Participants were excluded if they had a new onset (within the past 6 months) of an organic gastrointestinal disorder involving the lower gastrointestinal tract (i.e., inflammatory bowel disease, microscopic colitis, collagenous colitis, colonic strictures or malignancy).

### Measures

#### Participant characteristics

A demographic questionnaire was used to collect information such as age, gender, race/ethnicity, highest education level achieved, current employment status, length of time since diagnosis, and medications taken for the treatment of depression and/or anxiety.

#### Illness representations

The Revised Illness Perception Questionnaire (IPQ-R) was used to quantify the components of illness representation. The subscales included were identity, consequences, timeline-acute/chronic, timeline cyclical, treatment and personal control, illness coherence, emotional representation, and cause. The items were rated on a 5-point scale except for the identity scale. The identity scale included 14 commonly experienced symptoms. The participant was asked to indicate whether they experienced any of the symptoms since their IBS diagnosis and whether they believe that the symptoms listed were related to their IBS. The cause scale lists 18 potential causes associated with IBS. Good reliability and validity were demonstrated in all subscales [15].

#### Health-related quality of life

The Irritable Bowel Syndrome-Quality of Life (IBS-QOL) questionnaire was used to quantify the level of health-related quality of life, including global and subscale measurement. The 34 items were rated on a 5-point scale that ranged from “not at all” to “extremely or a great deal,” where higher scores imply better quality of life. Internal consistency and reliability were demonstrated [16].

#### Catastrophizing

The catastrophizing subscale of the Coping Strategies Questionnaire was used and includes six items that reflected the participant’s feelings of hopelessness and worsening pain expectation. Participants were classified as catastrophizers and non-catastrophizers, using median split to ensure that categorization was balanced and accurately reflected the true “split” for this sample [17, 18]. Good reliability and validity have been shown in chronic pain patients in addition to IBS patients using the Coping Strategies Questionnaire subscale [19].

#### Psychological distress

The Brief Symptom Inventory 18 (BSI-18) was used to quantify the levels of anxiety, depression, and somatization over the previous 7 days. Items were rated on a 5-point scale that ranged from “not at all” to “extremely” where greater scores imply higher levels of anxiety, depression, and somatization. The BSI-18 has good internal consistency and validity [20, 21].

### Average pain numeric rating scale

Abdominal pain intensity over the previous 7 days was rated on an 11-point numeric rating scale. Item anchors ranged from “no pain” to “worst pain possible,” where higher scores imply higher pain levels [22].

### Data analysis

Descriptive statistics of the participants were calculated and frequency tables (categorical variables) or measures of central tendency and spread (continuous variables) were reported. Due to the ordinal nature of the measures, Spearman correlations were used to measure the association between measures of interest. Because of the non-normal nature of the data, Wilcoxon Rank-Sum Tests were used to compare the scores for participants who were categorized as catastrophizers and non-catastrophizers. Chi-square analyses and Fisher’s Exact test were used to evaluate the associations between categorical variables. All assumptions were checked; if the Chi-square assumption was not met, data were dichotomized in order to use Fisher’s Exact test for categorical data. Only independent variables significantly associated with the HRQOL at the bivariate level of analysis were included as predictor variables in the subsequent multivariate analysis. Multiple linear regression analysis was conducted to predict the HRQOL using catastrophizing and psychosocial variables after controlling for age, gender, and pain. All analyses were conducted with SAS® software, version 9.4 (Copyright © 2012 SAS Institute Inc.) or Statistical Package for Social Sciences for Windows, version 23.0 (IBM SPSS version 23.0, Chicago, Illinois).

## Results

### Participant characteristics

A total of 101 IBS patients participated. The mean age was 42.08 years (SD = 5.84; age range 30–50 years). Participants were predominantly female (78.22%), Caucasian (96.04%), and had at least 4 years of college (Bachelor’s degree or higher 49.50%) or some college/vocational or technical education (23.76%). For the entire study cohort, there was no significant difference in age, gender, marital status (69% married), race, or employment (58% full-time) between catastrophizers *(N* = *52)* and non-catastrophizers *(N* = *49)*. Participant-reported pain levels in the past week (catastrophizers median 5.0 vs. non-catastrophizers median 3.0) were statistically different (*p* ≤ 0.0001; Wilcoxon Rank-Sum Test). Fifty-five percent of the overall participants took medication for the treatment of depression, anxiety, or both and 60.39% had been diagnosed with IBS for 5 or more years.

### Catastrophizing

Group categorization of catastrophizers and non-catastrophizers used the median split catastrophizing score of 7.00 (range = 0–36). The median split resulted in a group of catastrophizers (n = 52) and non-catastrophizers (n = 49).

### Illness representations

#### Emotional representations and consequences

**Fig. 1:**
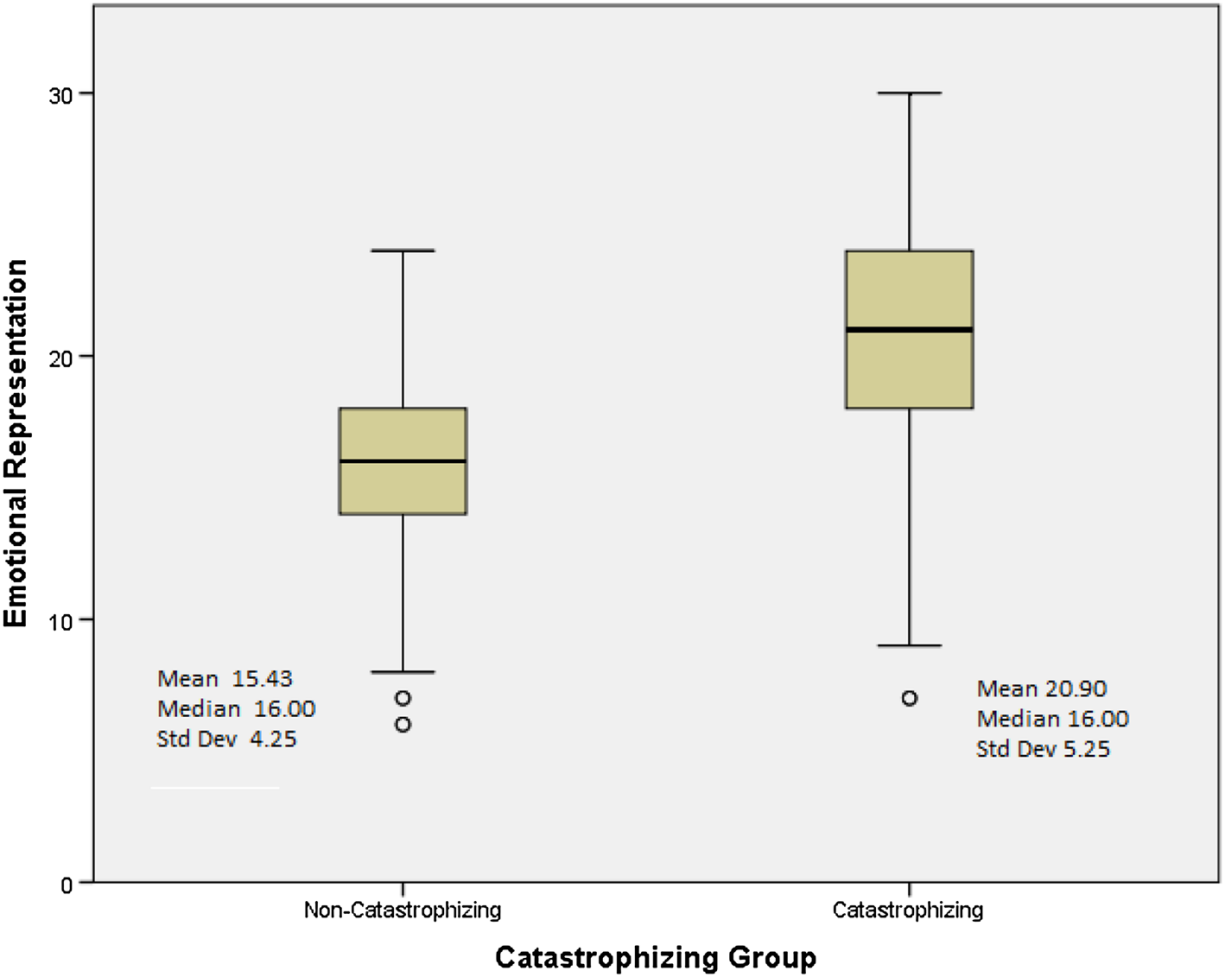
Emotional representation by catastrophizing category with mean, median, and standard deviation of emotional representation for each group (*p* = 0.001)

**Fig. 2:**
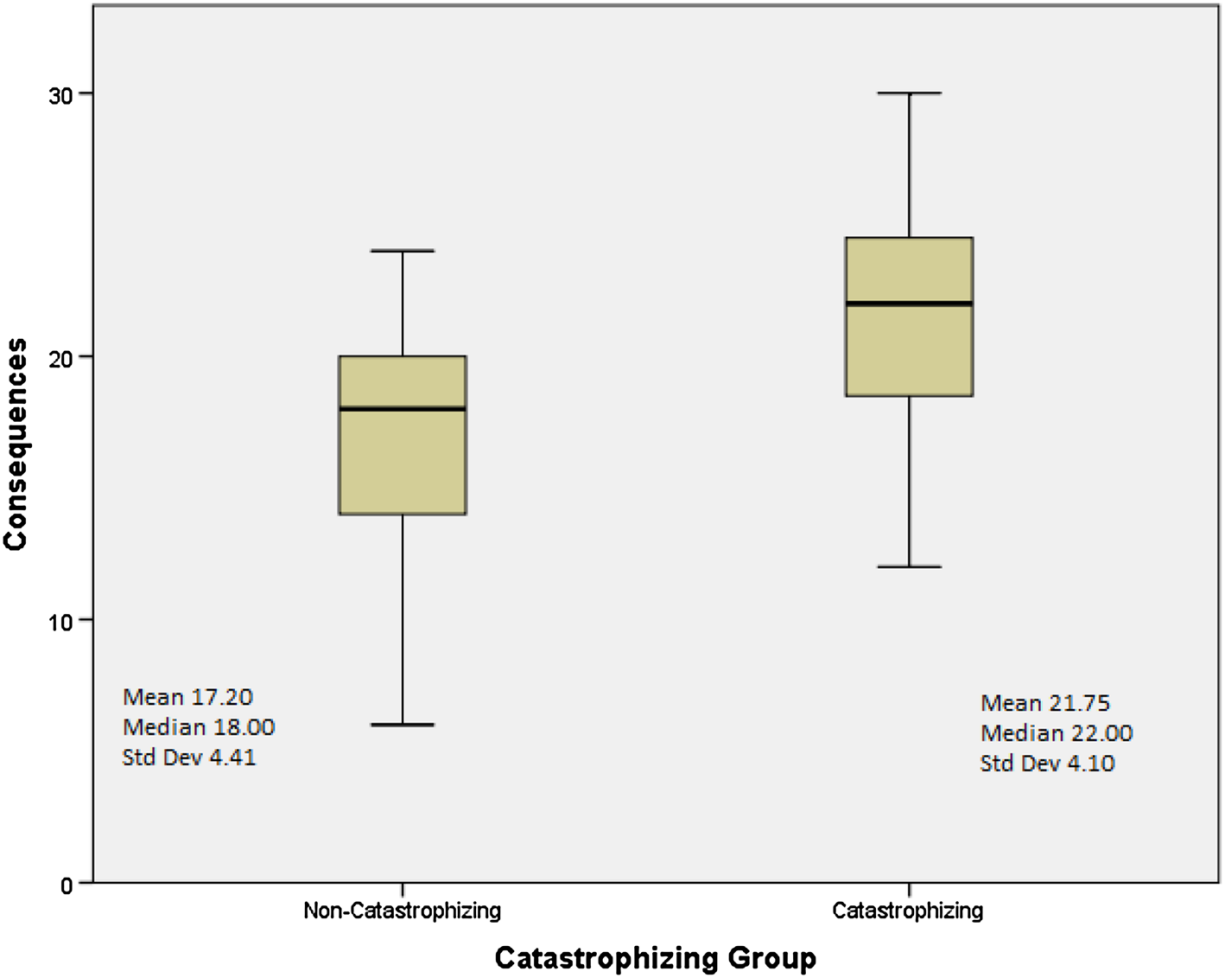
Consequence score by catastrophizing category with mean, median, and standard deviation of consequence for each group (*p* = 0.0002)

Emotional representation and consequences were significantly higher in catastrophizers as compared to non-catastrophizers (Figs. 1, 2). The remaining illness representations (timeline-acute/chronic/cyclical, cure/control-personal/treatment, identity, illness coherence) did not differ significantly between catastrophizers and non-catastrophizers.

#### Causal representation

The overall cohort most commonly reported causal attributions were “risk factors” (hereditary, diet, poor medical care, own behavior, aging, smoking, or alcohol) (53%) and psychological attributions (46%). Only one participant identified immunity (germ/virus, pollution in the environment or altered immunity) as a cause of their IBS. Catastrophizers did not differ significantly from non-catastrophizers on their causal reporting.

#### IBS quality of life

Overall, participants reported poor HRQOL (*M* = 63.32, median = 63.97, range 0–100). However, the quality-of-life scores were significantly lower in catastrophizers (*M* = 48.98; median = 48.90; *p* ≤ 0.001) as compared to non-catastrophizers (*M* = 78.53, median = 80.88). Furthermore, all subscales significantly differed between the groups (Table 1). Catastrophizers were noted to endorse lower levels of subscale values, implying that IBS had a significant negative impact on their physical and psychological well-being as compared to non-catastrophizers.

**Table 1:** Description of the measures by catastrophizing category (mean, median, standard deviation, and significance)

Catastrophizing category	Variable	Mean	Median	SD	*p* value
Catastrophizers	Total HRQOL	48.98	48.89	20.18	≤0.0001
Non-catastrophizers	Total HRQOL	78.53	80.88	14.08	
HRQOL subscales					
Catastrophizers	Body image	48.20	50.00	23.00	≤0.0001
Non-catastrophizers	Body image	71.30	75.00	21.91	
Catastrophizers	Dysphoria	49.34	53.13	25.47	≤ 0.0001
Non-catastrophizers	Dysphoria	84.89	90.63	13.92	
Catastrophizers	Food avoidance	35.26	33.33	30.53	≤0.0001
Non-catastrophizers	Food avoidance	62.93	66.67	28.33	
Catastrophizers	Health-worry	50.16	54.17	22.66	≤0.0001
Non-catastrophizers	Health-worry	77.38	83.33	19.10	
Catastrophizers	Interference with activity	42.72	42.86	23.04	≤0.0001
Non-catastrophizers	Interference with activity	75.51	75.00	18.59	
Catastrophizers	Relationships	62.98	66.67	24.33	≤0.0001
Non-catastrophizers	Relationships	86.90	91.67	14.63	
Catastrophizers	Sexual	54.57	62.50	34.48	≤0.0001
Non-catastrophizers	Sexual	79.85	100.00	26.12	
Catastrophizers	Social reaction	56.13	59.38	29.72	≤0.0001
Non-catastrophizers	Social reaction	83.93	87.50	15.78	
BSI-18					
Catastrophizers	Global severity index	21.35	21.00	13.82	≤0.001
Non-catastrophizers	Global severity index	6.76	4.00	8.00	
BSI-18 subscales					
Catastrophizers	Somatization	8.19	6.00	6.07	≤0.001
Non-catastrophizers	Somatization	3.51	2.00	4.19	
Catastrophizers	Anxiety	7.35	7.50	5.58	≤0.001
Non-catastrophizers	Anxiety	1.96	1.00	2.58	
Catastrophizers	Depression	5.81	4.00	5.57	≤0.001
Non-catastrophizers	Depression	1.29	0.00	2.17	

*HRQOL* health-related quality of life as measured by the IBS-QOL (Irritable Bowel Syndrome-Quality of Life) condition-specific measure, BSI—18 Brief Symptom Inventory-18

#### Psychological distress

The BSI-18 global severity index scores were significantly higher in catastrophizers (*M* = 21.35, median = 21.00; SD = 13.82; range = 0–68; *p* ≤ 0.001) than in non-catastrophizers (*M* = 6.76, median = 4.00; SD = 8.00; range = 0–13; *p* ≤ 0.001). When the distribution of the three psychological distress categories (anxiety, depression, and somatization) was compared between catastrophizers and non-catastrophizers, the difference was highly significant (*p* ≤ 0.001; Table 1).

#### Correlations

Overall, catastrophizing was positively associated with the identity, consequences, and emotional representations and negatively associated with HRQOL total and subscales (Table 2). In addition, total HRQOL was negatively associated with psychological distress variables: anxiety (*r* = −.66; *p* ≤ 0.001), depression (*r* = −.60; *p* ≤ 0.001), somatization (*r* = −.51; *p* ≤ 0.001), and global severity index (*r* = −.68; *p* ≤ 0.001), as well as pain experienced in the previous week (*r* = −.54; *p* ≤ 0.001).

**Table 2:** Spearman correlations among health-related quality of life (total and subscales), catastrophizing coping style, and illness representation components (*N* = 101)

	HRQOL	CCS	1	2	3	4	5	6	7	8
HRQOL total			−0.47**	−0.72**	−0.34**	0.39**	0.39**	0.40**	−0.18	−0.71**
Catastrophizing coping style	−0.76**		0.39**	0.54**	0.27**	−0.39**	−0.35**	−0.33**	0.20	0.65**
HRQOL subscales										
Body image		−0.54**	−0.40**	−0.42*	NS	−0.28*	−0.28*	0.32**	0.19*	−0.52**
Dysphoria		− 0.73**	−0.26**	−0.66**	0.32**	0.44**	−0.36**	0.41**	NS	−0.78**
Food avoidance		− 0.51**	− 0.26*	− 0.52**	− 0.20*	NS	NS	0.23*	− 0.25*	− 0.48**
Health-worry		−0.64**	−0.40**	−0.47**	NS	0.24*	0.38**	0.32**	−0.21*	−0.54**
Interference with activity		−0.69**	−0.37**	−0.64**	−0.22**	0.31**	0.30*	0.48**	−0.25*	−0.59**
Relationships		−0.56**	−0.28*	−0.65**	−0.29**	0.32**	0.33**	0.34**	NS	−0.61**
Sexual		−0.46**	−0.39**	−0.60**	−0.23*	0.31**	0.35**	0.25*	NS	−0.47**
Social reaction		−0.55**	−0.25*	−0.57**	−0.29*	0.22*	0.27*	0.23*	NS	−0.60**
Illness representation components										
1. Identity				0.44**	0.15	−0.25*	−0.24*	−0.27**	NS	0.35**
2. Consequences					0.43**	−0.41**	−0.58**	−0.32**	NS	0.65**
3. Timeline, acute/chronic						−0.30**	−0.44**	−0.23**	NS	0.28**
4. Personal control							0.61**	0.54**	NS	−0.44**
5. Treatment control								0.35**	NS	−0.43**
6. Illness coherence									NS	−0.38**
7. Timeline cyclical										NS
8. Emotional representation										

HRQOL health-related quality of life; CCS catastrophizing coping style; NS non-significant; 1 identity; 2 consequences; 3 timeline, acute/chronic; 4 personal control; 5 treatment control; 6 illness coherence; 7 timeline cyclical; 8 emotional representation

*p ≤ 0.05, **p ≤ 0.001

#### Multiple regression

Multiple linear regression of the variables influencing HRQOL in IBS included six independent predictor variables (catastrophizing, anxiety, somatization, depression, the interaction between anxiety and somatization, and anxiety and depression) with total HRQOL scores from the IBS-QOL as the dependent variable (Table 3). The resultant analysis revealed that catastrophizing, anxiety, somatization, depression, and the interaction of anxiety and somatization, and anxiety and depression each independently predicted poorer HRQOL independent of covariates (age, sex, and pain). The model had an *R* ^2^ of 0.79, suggesting that over three quarters of the observed variability in HRQOL was accounted for by this model. Catastrophizing was the single greatest predictor of HRQOL accounting for 32% of total HRQOL variability. Psychological distress variables together accounted for a small amount of variability in participants’ HRQOL (15%), with anxiety accounting for the greatest amount of variance, 8%, and the interaction of anxiety and somatization, and anxiety and depression contributing 3 and 4%, respectively.

**Table 3:** Multivariate regression analyses predicting health-related quality of life by the psychosocial predictor variables controlling for demographic variables in adults with irritable bowel syndrome

Predictor variables	*β*	95% CI for *β*		Semi-partial eta-square
Age	−0.1179078	−0.5165177	0.2807021	0.0119
Sex (Female)	−7.2099430	−12.5963252	−1.8235608	0.0095
Pain	−2.3974491	−3.6435407	−1.1513575	0.3131
Catastrophizing	−1.1027754	−1.5275834	−0.6779674	0.3149
Anxiety	−1.2723519	−2.2751593	−0.2695445	0.0794
Somatization	1.5202876	0.6005868	2.4399884	0.0000
Depression	−2.4499983	−3.6279140	−1.2720826	0.0003
Anxiety*somatization	−0.1659669	−0.2506989	−0.0812349	0.0353
Anxiety*depression	0.2228991	0.1355720	0.3102263	0.0258
*R* ^*2*^ full model	0.79***	NA		

*β*standardized regression coefficients (beta weights), *R* ^*2*^ percentage of variability in the dependent variable (HRQOL mean) explained in the model

*p < 0.05, **p < 0.01, ***p < 0.0001

## Discussion

The main focus of this study was to examine whether the use of catastrophizing coping impacted the health-related quality of life, psychological distress, and illness perceptions of adults who suffer from IBS. Catastrophizing, an important psychological concept with limited previous research in IBS, was found to be significantly endorsed in this sample. Furthermore, individuals who catastrophized reported worse health-related quality of life, higher psychological distress, and perceived more somatic symptoms, worse consequences, and more severe emotional impact as compared to those participants who did not catastrophize.

Catastrophizing was positively associated with consequences, emotional, identity, and timeline representations and negatively associated with health-related quality of life, control, and coherence representations. These findings indicate that individuals who catastrophized associated a greater number of symptoms with their IBS and categorized their IBS as more chronic in nature and perceived worse consequences and a worse emotional impact. Alternatively, those reporting greater personal and treatment control and greater understanding of IBS were associated with a better health-related quality of life.

The role of catastrophizing in illness perceptions has not previously been reported in an IBS population. Previous research has confirmed the association between catastrophizing and depression, and pain severity [6, 23–25]. In addition, catastrophizing has been found to be a mediator between psychological distress and pain [26] and attachment style and symptom severity [27]. Our findings further highlight the importance of this cognitive coping style frequently associated with IBS.

Additionally, it is important to mention that we found psychological distress (anxiety, depression, and somatization) was significantly associated with impairment of HRQOL. The main effects and interaction between anxiety and depression explained a third of the variation that catastrophizing or pain explained. Of the psychological distress variables, anxiety was the greatest predictor of HRQOL impairment. The literature has established that individuals with IBS often experience comorbid psychological distress [5, 11, 28–30] and psychological distress is consistently found to negatively impact patient’s quality of life as measured by physical and mental functioning [12]. In addition, van Tilburg et al. (2013) have found that psychological distress (anxiety) relation to IBS symptom severity was mediated by catastrophizing [5]. It is important to keep in mind the impact psychological factors have on individuals who suffer from IBS, especially with regard to their HRQOL. However, given the results of this study, it may be prudent to also consider the maladaptive coping skills, in particular catastrophizing coping, as the findings of our study suggest that maladaptive coping in addition to psychological distress may play an important role in IBS outcomes.

Medical treatment modalities in IBS have had limited success [31, 32]. The influence catastrophizing has on the well-being of individuals with IBS suggests directions for the design of psychosocial interventions targeted toward catastrophizing as a possible means to positively impact behavior and functioning. Influencing outcomes positively with Cognitive Behavior Therapy (CBT) has been reported in the pain literature [33, 34], as well as in IBS [35]. However, varying duration, intervention methods, effect sizes, and diversity of CBT formats contribute to treatment inconsistencies and varying benefits. Thorn and colleagues [33] have developed a CBT program that centers on the diminishment of catastrophizing. The authors report a clinical observation that those with chronic pain respond to adaptive coping training only after they become aware of their catastrophizing. Research to assess and direct treatment specifically focused at reducing catastrophizing resulting in positive outcomes is needed. Such research would provide healthcare providers with greater insight into the mechanisms of change.

In addition to focusing treatment regimens on catastrophizing, addressing illness representations also appears to be a pertinent variable on which to focus. Illness representations have been noted to impact outcomes in such disorders as heart disease [36], rheumatoid arthritis [37], cancer [38, 39], and limited reports in IBS [3, 4]. There is persistent evidence for the theoretical predictable relations between illness perceptions, coping, and outcomes across these studies. Assessment and integration of both catastrophizing and illness perceptions into the management of individuals who suffer with IBS may maximize health outcomes.

There are limitations to this study that need to be acknowledged. First, this study relied on patient-reported measures. Self-reported responses have been noted as potential limitation to studies [40]. There is a risk that a participant may be unable to remember information, such as description of personal views, feelings, distress level, and way of thinking. However, specific to this study it is important to note that these are the participant-reported perceptions and in contrast may actually have strengthened this study by further validating the experience of IBS. Second, the sample was relatively small and composed primarily of women from specialty gastrointestinal practices. Women are disproportionately affected with IBS [41]. The high distribution of women in our sample may be due in part to this disproportion in prevalence. The results may not generalize to males and/or non-gastrointestinal specialty practice patients. Given the sample size, results can only favor a trend that necessitates confirmation in larger sample size studies. In addition, the sample consisted of a significant number of participants that catastrophized compared to non-catastrophizers. As noted in the past research of individuals who suffer from IBS, there is a tendency to catastrophize [5, 42]. It is unclear if this sample is representative of this tendency as there is no current literature quantifying catastrophizing frequency in an IBS population. A final limitation is that our data were cross-sectional in nature and as a result cannot speak to the direction, if any, of causal effects.

## Conclusion

We provide evidence for the importance of assessing catastrophizing in individuals with IBS. Development of psychotherapeutic treatment strategies designed specifically for those with IBS with the focus to reduce their catastrophizing coping through reconceptualization of their IBS by altering their thoughts, beliefs, and behaviors may decrease the use of maladaptive coping strategies such as catastrophizing and result in the enhancement of their HRQOL. In addition, considering their illness perceptions has the potential to maximize health outcomes in those who suffer from irritable bowel syndrome. Lastly, psychological distress continues to remain an important factor in IBS management.
